# Headache in epilepsy: prevalence and clinical features

**DOI:** 10.1186/s10194-015-0556-y

**Published:** 2015-08-06

**Authors:** G Mainieri, S Cevoli, G Giannini, L Zummo, C Leta, M Broli, L Ferri, M Santucci, A Posar, P Avoni, P Cortelli, P Tinuper, Francesca Bisulli

**Affiliations:** IRCCS Istituto delle Scienze Neurologiche di Bologna, AUSL di Bologna, Bologna, Italy; Department of Biomedical and Neuromotor Sciences, University of Bologna, Bellaria Hospital, Via Altura, 3 – 40139, Bologna, Italy; Department of Experimental Biomedicine and Clinical Neuroscience, University of Palermo (BIONEC), Palermo, Italy

**Keywords:** Headache, Epilepsy, Migraine, Pre-ictal headache, Post-ictal headache

## Abstract

**Background:**

Headache and epilepsy are two relatively common neurological disorders and their relationship is still a matter of debate. Our aim was to estimate the prevalence and clinical features of inter-ictal (inter-IH) and peri-ictal headache (peri-IH) in patients with epilepsy.

**Methods:**

All patients aged ≥ 17 years referring to our tertiary Epilepsy Centre were consecutively recruited from March to May 2011 and from March to July 2012. They underwent a semi-structured interview including the International Classification Headache Disorders (ICHD-II) criteria to diagnose the lifetime occurrence of headache.χ^2^-test, t-test and Mann–Whitney test were used to compare clinical variables in patients with and without inter-IH and peri-IH.

**Results:**

Out of 388 enrolled patients 48.5 % had inter-IH: migraine in 26.3 %, tension-type headache (TTH) in 19.1 %, other primary headaches in 3.1 %. Peri-IH was observed in 23.7 %: pre-ictally in 6.7 %, ictally in 0.8 % and post-ictally in 19.1 %. Comparing patients with inter-ictal migraine (102), inter-ictal TTH (74) and without inter-IH (200), we found that pre-ictal headache (pre-IH) was significantly represented only in migraineurs (OR 3.54, 95 % CI 1.88-6.66, P < 0.001). Post-ictal headache (post-IH) was significantly associated with both migraineurs (OR 2.60, 95 % CI 1.85-3.64, P < 0.001) and TTH patients (OR 2.05, 95 % CI 1.41-2.98, P < 0.001). Moreover, post-IH was significantly associated with antiepileptic polytherapy (P < 0.001), high seizure frequency (P = 0.002) and tonic-clonic seizures (P = 0.043).

**Conclusions:**

Migraine was the most represented type of headache in patients with epilepsy. Migraineurs are more prone to develop pre-IH, while patients with any inter-IH (migraine or TTH) are predisposed to manifest a post-IH after seizures.

## Background

Epilepsy and primary headache disorders affect individuals of all ages worldwide. Several studies have been performed to attest if there is a relationship between the two conditions, in order to verify the existence of a causal association or if the two disorders can occur in the same individual by chance. In the last century Gowers first advanced the clinical hypothesis of a relationship between epilepsy and migraine [1] since the two conditions show a well-recognized clinical, pathophysiological and therapeutic overlap [2]. Studies on the association between epilepsy and other types of primary headache are difficult to perform as tension-type headache (TTH) is extremely common in the general population [3] whereas cluster headache is very rare [4]. For this reason most studies analyzing the prevalence of headache in patients with epilepsy focused only on migraine and results remain controversial (Table 1) [5–21].

**Table 1:** Literature prevalence of migraine in patients with epilepsy

Authors	Sample	M/F	Age	Methods	Results
	N of pts		yrs		Migraine
Schon and Blau 1987 [5]	100	39/61	32 mean	Interview	9 %
Ottman and Lipton 1994 [6]	1948	40 %/60 %	≥18	Structured telephone interviews + medical records review for 60 % of probands	24 %
Ito and Shachter 1996 [7]	162	82/80	19-65 range	Questionnaires mailed to the subjects + medical records review	NA^a^
Ito et al. 1999 [8]	109	36/73	38 ± 12 mean^b^	Questionnaire + interview + medical records review	12.8 %
Velioglu and Ozmenoglu 1999[9]	412	212/200	15-70 range	Interview with a standardized questionnaire	14 %
Leniger et al. 2001 [10]	341	154/187	40 ± 15 mean	Interview with a standardized questionnaire	18.2 %
Karaali Savrun et al. 2002 [11]	135	80/55	≥10	Questionnaire administered to patients	14.8 %
Förderreuther et al. 2002 [12]	110	69/41	35.2 mean	Semi-standardized interview	10 %^b^
Ito et al. 2004 [13]	364	163/201	12-81 range	Structured interview with standardized questionnaire	8 %
Syvertsen et al. 2007 [14]	109	44/65	20-71 range	Questionnaire + semi-structured telephonic interview	20 %
Kwan et al. 2008 [15]	227	98/129	36.0 ± 11.3 mean	Interview with standardized questionnaire + seizures and headache diary over the 3-month observation period + final interview	6.6 %^b^
HELP Study Group 2010 [16]	597	348/249	≥13	Questionnaire at initial visit	12.4 %
Tonini et al. 2012 [17]	492^c^	154/338	≥18	Direct interview with questionnaire	18.3 %^b^
Duchaczek et al. 2012 [18]	201	106/95	≥18	Semi-structured interview	11 %
Winawer et al. 2013 [19]	730^d^	285/445	≥12	Telephone or in-person interview + medical record abstraction	25.2 %^e^
Gameleira et al. 2013 [20]	304	141/163	4-88 range	Patients evaluated at the epilepsy clinic	32.9 %^f^
Wang et al. 2014 [21]	1109	607/502	≥18	Self-administered questionnaire + standardized semi-structured telephone interview	12.53 %

N, number; pts, patients; M, males; F, females; yrs, years; NA, not available

^a^a prevalence of inter-ictal migraine is not clearly identifiable; the authors report a prevalence of inter-ictal headache in 64 % of patients, approximately a half of them with a pounding quality and almost 70 % of them often accompanied by nausea and/or vomiting, photophobia or phonophobia

^b^calculated by the authors

^c^this multicenter study involved 1167 patients from epilepsy and headache centers, we considered only patients with epilepsy

^d^371 probands, 231 siblings, 128 parents: all with epilepsy; ^e^23.5 % probands, 22.5 % siblings, 35.2 % parents

^f^the authors of the study does not distinguish between inter-ictal migraine and post-ictal headache with migrainous features

According to its temporal relationship with epileptic seizures, headache can be classified as inter-ictal (inter-IH) or peri-ictal (peri-IH). Inter-IH is not temporally related to seizures, whereas peri-IH manifests in their time frame (pre-ictally, ictally, post-ictally) [7, 8]. Literature data on the relation between inter-IH (in particular migraine) and peri-IH are controversial owing to the methodological heterogeneity of previous studies [5–21]. Moreover, in the context of seizure-related headaches, entities identified as “migralepsy” or “epileptic headache” are still matter of discussion [22].

The aim of this study was to estimate the prevalence of headache in adult patients with epilepsy, describing its clinical features and temporal relationship with seizure occurrence.

## Methods

The institutional review board of the IRCCS Institute of Neurological Sciences of Bologna approved the project. Clinical investigations have been carried out in accordance with the Helsinki Declaration adopted by the 18th World Medical Assembly in Helsinki, in 1964, as last amended by the World Medical Assembly.

### Study design and participants

This is a cross-sectional study conducted at the outpatient clinic of our tertiary Epilepsy Center between March and May 2011 and March and July 2012. Patients aged ≥ 17 years were consecutively asked to participate in the study and a self-report form was administered to those who accepted. This form dichotomously ruled out patients who reported a lifetime presence of headache and patients who had never suffered from headache. If patients confirmed the occurrence of headache, trained physicians (GM, CL, LF), blinded to the patient’s diagnosis, conducted a semi-structured interview characterizing the type of inter-IH and peri-IH, if present. Headache data were revised by headache experts (SC, GG, PC), who validated the diagnosis according to ICHD-II criteria [23]. Expert epileptologists (FB, PT, PA, MS) classified epileptic seizures and syndromes according to the 2010 International League Against Epilepsy (ILAE) Commission report [24].

All patients with a diagnosis of epilepsy were included in the study. We excluded patients who had arrived for a first visit and proved not to be affected by epilepsy (i.e. psychogenic non-epileptic seizures, sleep disorders, syncope, dystonia), patients with a doubtful epilepsy diagnosis, patients who had only a single seizure, and patients with a severe mental retardation.

### Data collection

The self-report form administered contained socio-demographic data and a preliminary question regarding the lifetime presence of headache. Patients who answered affirmatively also had to indicate their age at headache onset, headache in the previous three months, the frequency of attacks and the use of analgesics in a month. On the same form, patients who reported headache also answered the already validated ID migraine, a three-item instrument for migraine screening, and then underwent an ad hoc semi-structured interview with trained physicians concerning inter-IH and peri-IH. The formulation of this semi-structured interview was the product of a collaboration between the Epilepsy and Headache Centers. The clinical data collected to evaluate inter-IH concerned headache in other family members, age at headache onset, lateralization of headache, quality of pain, duration, intensity, frequency in a month, worsening with physical activity, use of analgesic treatment, associated symptoms like photophobia, phonophobia, nausea, vomiting, and presence of aura. For patients who presented peri-IH, the interview also included questions on timing of onset (pre-ictal, ictal or post-ictal headaches), duration, and features of peri-IH. Finally expert epileptologists filled out the last section of the form reviewing patients’ clinical records and collecting data on epilepsy syndrome, seizure types, frequency of seizures, epilepsy etiology, age at epilepsy onset, disease duration, current antiepileptic medications, and photosensitivity. The interview had previously been tested on a series of 50 consecutively recruited patients with epilepsy, showing that the diagnosis gathered from the semi-structured interview correlated with that of the headache experts.

### Definitions and classifications

According to the temporal relationship with seizures, peri-IH was divided into pre-ictal headache (pre-IH), ictal and post-ictal headache (post-IH). Pre-IH was defined as appearing within 24 h before the seizure [18, 22, 25]. Ictal headache was present exclusively during the seizure [22]. Post-IH was defined according to the ICHD-II as a “headache which develops within 3 h following a partial or generalized seizure and resolves within 72 h after the seizure” [23]. We collected data on the lifelong presence of headache and verified if headache attacks had occurred in the three months prior to the interview. We defined as “inter-ictal headache” all headaches that manifested within a time period of the epileptic disease and whose attacks were not temporally related to an epileptic seizure. According to ICHD-II criteria [23], inter-IH was divided into migraine (with or without aura), probable migraine, TTH, probable TTH, cluster headache, and other primary headaches. Secondary headaches in structural epilepsies were ruled out by means of imaging studies and were not considered in our analyses.

### Statistical analysis

Clinical variables in patients with inter-ictal migraine, inter-ictal TTH and without inter-IH were analyzed. In addition we compared clinical features between patients with post-IH and those without post-IH and between patients with pre-IH and those without pre-IH. We performed χ^2^-test and t-test to compare categorical variables. T-test and Mann–Whitney test were performed to evaluate continuous variables with a symmetrical and asymmetrical distribution respectively. A logistic regression model was used to calculate OR and a 95 % confidence interval CI to assess the association between dependent and independent variables. Adjustment for the possible effect of confounding variables such as age, sex and migraine prophylactic therapy when appropriate was performed through a multivariable-adjusted logistic regression analysis. Statistical significance was set at p value < 0.05. Statistical analyses were performed with STATA® version 12.0.

## Results

### Study population

**Fig. 1:**
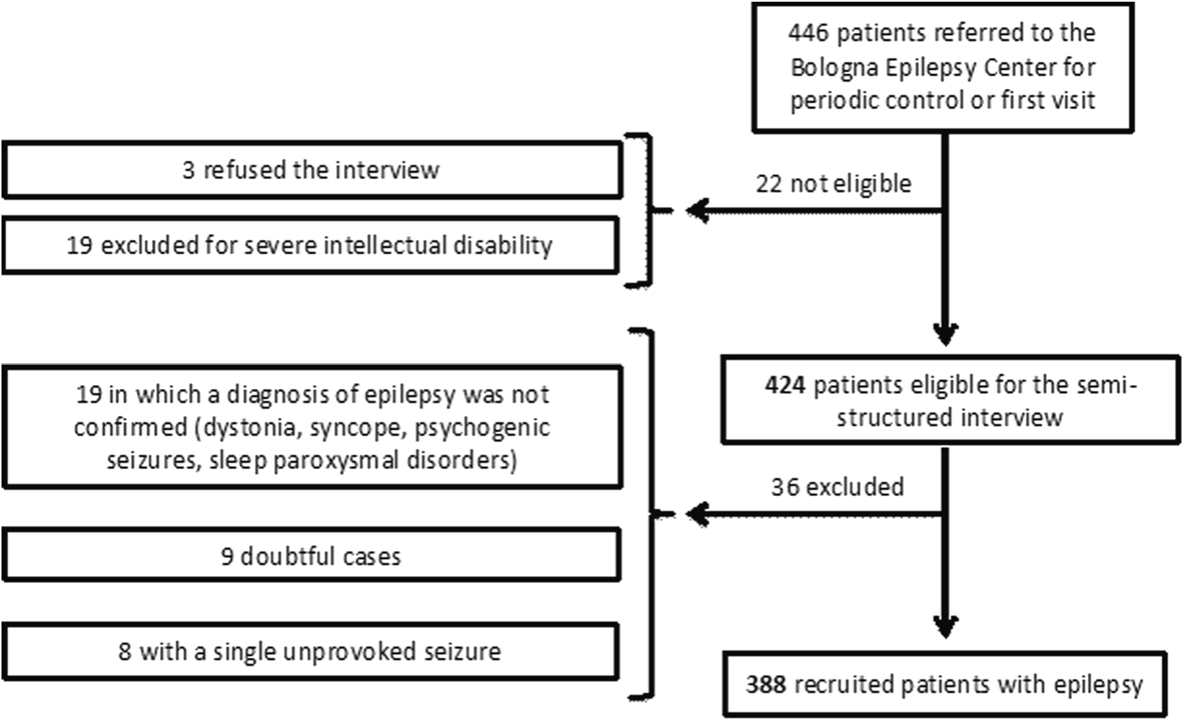
Flow diagram of enrolled patients. Patients recruited from a pool of 454 subjects referred to the Bologna Epilepsy Center. Thirty-six patients excluded after medical review of their clinical records that did not confirm a diagnosis of epilepsy

The flow diagram of the study is illustrated in Fig. 1 and the main features of the population in Table 2. Out of the original pool of 446 outpatients attending the Epilepsy Center we included in the study 388 cases (209 female, 53.9 %) with a confirmed diagnosis of epilepsy. The mean age of patients at the interview was 41.25 ± 15.70 years (median 39, range 17–84). One hundred and one patients had generalized epilepsy (26.0 %), 77 with a genetic etiology and 24 with a structural/metabolic or unknown etiology. Two hundred and eighty patients had focal epilepsy (72.2 %), four with a genetic etiology and 276 with a structural/metabolic or unknown etiology. Seven patients had unclassified epilepsy (1.8 %). The median age at epilepsy onset was 15 years (interquartile range 8–23.5) and the median epilepsy duration at the interview was 20.5 years (interquartile range 11–32). One hundred and thirty-four patients (34.5 %) had sporadic seizures (few episodes per year), 132 (34.0 %) had been seizure-free for at least two years at the interview while 119 (30.7 %) had monthly/daily or multidaily seizures (in three cases frequency could not be assessed). One hundred and eighty-five patients (47.7 %) were taking antiepileptic monotherapy, 188 (48.4 %) received polytherapy (≥2 AEDs), and 15 (3.9 %) had no therapy at the interview. In the latter group therapy had been withdrawn in 13 cases 4.5 years on average before the interview, after many years of seizure freedom one patient had a recent disease history with only two seizures and was not on any therapy at the interview while another had sporadic seizures and was not on therapy of his own accord.

**Table 2:** Clinical features of the study population

	Total	Males	Females
Sample	388	179 (46.13)	209 (53.87)
Age *(mean ± SD)*	41.25 ± 15.70	41.92 ± 16.32	40.68 ± 15.15
Age at epilepsy onset *(med; p25-p75)*	15; 8–23.5	16; 7-26	14; 9-22
Epilepsy duration *(med; p25-p75)*	20.5; 11-32	20; 10-31	21; 11-32
Epilepsy			
Generalized	101 (26.03)	46 (25.70)	55 (26.32)
Focal	280 (72.16)	129 (72.07)	151 (72.25)
Unclassified	7 (1.80)	4 (2.23)	3 (1.43)
Frequency of seizures at observation			
Sporadic	134 (34.54)	59 (32.96)	75 (35.89)
Monthly/daily	119 (30.67)	49 (27.37)	70 (33.49)
Seizure-free	132 (34.02)	69 (38.55)	63 (30.14)
AED therapy			
Monotherapy	185 (47.68)	91 (50.84)	94 (44.98)
Polytherapy	188 (48.45)	82 (45.81)	106 (50.72)
No therapy	15 (3.87)	6 (3.35)	9 (4.31)
Photosensitivity	23 (5.93)	10 (5.59)	13 (6.22)
Inter-IH	188 (48.45)	68 (37.99)	120 (57.42)
Migraine without aura	80 (20.62)	25 (13.97)	55 (26.32)
Migraine with aura	6 (1.55)	4 (2.23)	2 (0.96)
Probable migraine	16 (4.12)	5 (2.79)	11 (5.26)
TTH	72 (18.56)	27 (15.08)	45 (21.53)
Probable TTH	2 (0.52)	1 (0.56)	1 (0.48)
Cluster headache	2 (0.52)	0 (0.00)	2 (0.96)
Peri-IH	92 (23.71)	34 (18.99)	58 (27.75)
Pre-ictal headache	26 (6.70)	6 (3.35)	20 (9.57)
Ictal headache	3 (0.77)	0 (0.00)	3 (1.44)
Post-ictal headache	74 (19.07)	30 (16.76)	44 (21.05)

N, number; SD, standard deviation; med, median; p25-p75, 25^th^ and 75^th^ percentile; AED, anti-epileptic drug; Inter-IH, inter-ictal headache; TTH, tension-type headache; Peri-IH, peri-ictal headache. Figures given as N (%), unless otherwise stated

### Headache

**Fig. 2:**
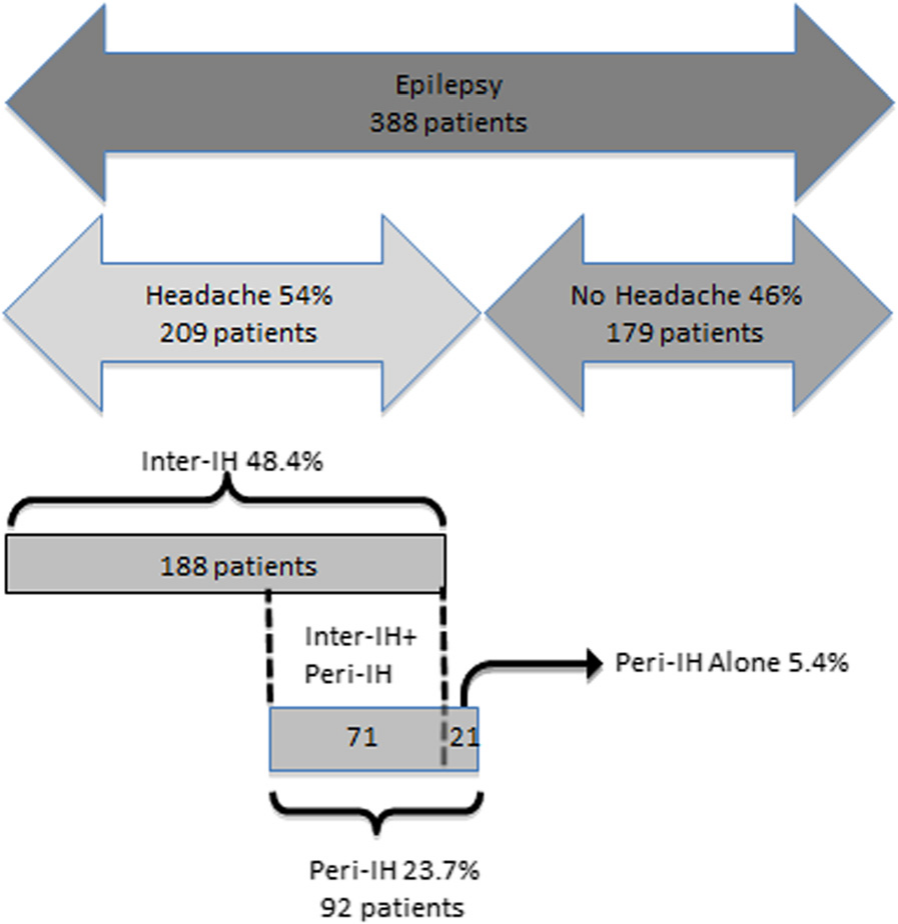
Flow chart describing patients presenting headache. This subgroup is further divided into patients with inter-ictal headache and patients with peri-ictal headache, with an overlapping group of individuals who presented both conditions. Inter-IH, inter-ictal headache; Peri-IH, peri-ictal headache

Overall 209 patients (53.9 %) reported a lifetime occurrence of headache: 188 had inter-IH (48.4 %), while peri-IH occurred in 92 patients (23.7 %). Among them 71 patients (18.3 %) had an associated inter-IH, while 21 (5.4 %) only had headache related to the seizures (Fig. 2). The latter occurred before the seizure in three patients, during the seizure in another three cases and in the post-ictal period in 16 patients (one of them had an ictal headache which continued post-ictally). Out of the 188 patients with inter-IH, 35 (18.6 %) did not report headaches in the previous three months. According to ICHD-II criteria [23], patients with inter-IH were classified as follows: 102 patients had migraine (26.3 %), including six with aura (1.5 %) and 16 with probable migraine (4.1 %), 74 had TTH (19.1 %), including two with probable TTH (0.5 %), two patients had cluster headache (0.5 %), one patient had primary stabbing headache (0.03 %) and nine patients had unclassified headache (2.3 %).

According to its temporal relationship with seizure onset *peri-ictal headache* was distinguished as:

1. *Pre-ictal headache* present in 26 patients (*6.7 %*), with migrainous features in 16, with tension-type quality in five, other in five. Only in one of these patients did the attacks disclose the features of migraine with aura but did not present a strict temporal relationship (within one hour) with seizure onset. Nineteen patients (4.9 %) with pre-IH had also inter-ictal migraine (one with aura) while one had an unclassified headache. In most of these cases (16/20), pre-IH had migrainous features similar in quality to the habitual headache attack, whereas four patients were not able to characterize the features of their pre-IH. Another three patients (0.8 %) presenting pre-IH had an inter-ictal TTH that showed similar quality features to the habitual attacks in all cases. Three patients (0.8 %) not complaining of inter-IH only presented headache before seizures: it showed tension-type headache-like features in two patients, while one patient did not recall the features of his pre-IH.
2. *Ictal headache* in three cases (*0.8 %*), none of whom had an inter-IH. The first patient had a myoclonic epilepsy and referred a sense of head pressure lasting a few seconds, involving the whole head, in correspondence with myoclonic jerks. The second patient had a focal epilepsy of temporal origin of the right hemisphere and referred a very intense mostly frontal throbbing pain (undetermined side) a few seconds before the seizure. The third patient had a focal epilepsy of temporal origin of the left hemisphere and reported a headache starting during the seizure and often continuing in a post-IH. The headache showed a tightening quality of moderate to severe intensity not associated with autonomic symptoms, photophobia or phonophobia.
3. *Post-ictal headache* occurring in 74 patients (*19.1 %*) with the following features: migraine quality in 37, TTH-like in 30, other in seven. Thirty-eight patients (9.8 %) had an associated inter-ictal migraine (no patients with aura). Their post-IH in most cases (31/38) had migrainous features, in six cases it was described as TTH-like, while one patient did not recall the features of his post-IH. Twenty patients (5.1 %) had an inter-ictal TTH. Their post-IH showed similar TTH-like features in 16 (16/20) patients and migrainous features in two (2/20), while in two patients it was unclassified. Sixteen patients (4.1 %) manifested headache only after seizures with migrainous features in four (4/16) of them and TTH-like features in eight (8/16), whereas it could not be better characterized in the remaining four cases.

### Migraineurs vs TTH vs patients without inter-IH

We compared clinical features in patients with inter-ictal migraine, patients with inter-ictal TTH and patients who did not have any inter-IH (Table 3). Female sex was prevalent both in the migraineurs group (P < 0.001) and in the TTH group (P = 0.009), compared to patients without inter-IH. Migraineurs had a lower mean age than patients without inter-IH (P = 0.013). There were no significant differences in epilepsy syndrome, seizure frequency, therapy or photosensitivity among the three groups. After adjustment for age, sex and therapy, both groups of patients with inter-ictal migraine (OR 2.68, 95 % CI 1.96-3.64, P < 0.001) and TTH (OR 1.87, 95 % CI 1.33-2.63, P < 0.001) were significantly associated with the occurrence of peri-IH. Pre-IH was significantly associated only with patients with inter-ictal migraine, both compared to patients without inter-IH (OR 3.54, 95 % CI 1.88-6.66, P < 0.001) and to patients with TTH (OR 5.29, 95 % CI 1.50-18.68, P = 0.010). Post-IH occurred significantly in both groups of migraineurs (OR 2.60, 95 % CI 1.85-3.64, P < 0.001) and TTH patients (OR 2.09, 95 % CI 1.44-3.03, P < 0.001) compared with patients without inter-IH, while there was no statistical difference between them (OR 1.69, 95 % CI 0.86-3.31, P = 0.127).

**Table 3:** Comparison of clinical features between subgroups of patients with inter-ictal migraine, inter-ictal tension-type headache and patients without inter-ictal headache

	Inter-ictal Migraine (Group 1)	Inter-ictal TTH (Group 2)	No inter-IH (Group 3)	*p* value	OR (CI 95 %)	Group Comparison
Sample	102 (26.29)	74 (19.07)	200 (51.55)			
Males	34 (23.45)	28 (20.14)	111 (79.86)	<0.001;	-	1 vs 3;
Females	68 (43.31)	46 (34.07)	89 (65.93)	0.009		2 vs 3
Age *(mean ± SD)*	38.57 ± 13.73	40.34 ± 16.46	43.26 ± 16.27	0.013	-	1 vs 3
Age at epilepsy onset *(med; p25-p75)*	15; 10-21	15; 10-26	15; 6-25	0.877	-	-
Epilepsy duration *(med; p25-p75)*	19; 9-31	19; 10-28	21.5; 12-34	0.076	-	-
Epilepsy						
Generalized	27 (26.47)	19 (25.68)	51 (25.50)	0.855	-	-
Focal	73 (71.57)	55 (74.32)	144 (72.00)	0.937	-	-
Unclassified	2 (1.96)	0 (0.00)	5 (2.50)	0.768	-	-
Frequency of seizures at observation						
Sporadic	37 (36.27)	24 (32.43)	67 (33.50)	0.631	-	-
Monthly/daily	30 (29.41)	24 (32.43)	62 (31.00)	0.777	-	-
Seizure-free	34 (33.33)	26 (35.14)	69 (34.50)	0.840	-	-
Tonic-clonic seizures	73 (71.57)	48 (64.86)	113 (56.50)	0.011	1.39 (1.08 - 1.80)	1 vs 3
AED therapy						
Monotherapy	54 (52.94)	36 (48.65)	91 (45.50)	0.221	1.16 (0.91 - 1.47)	-
Polytherapy	43 (42.16)	37 (50.00)	101 (50.50)	0.170	0.85 (0.67 - 1.08)	-
No therapy	5 (4.90)	1 (1.35)	8 (4.00)	0.715	1.11 (0.63 - 1.97)	-
Photosensitivity	8 (7.84)	4 (5.41)	11 (5.50)	0.428	-	-
Peri-ictal headache	48 (47.06)	22 (29.73)	21 (10.50)	0.019^a^;	2.18 (1.14 - 4.19);	1 vs 2;
				<0.001^a^;	2.68 (1.96 - 3.64);	1 vs 3;
				<0.001^b^;	1.87 (1.33 - 2.63)	2 vs 3;
Pre-ictal headache	19 (18.63)	3 (4.05)	3 (1.50)	0.010^a^;	5.29 (1.50 - 18.68);	1 vs 2;
				<0.001^a^	3.54 (1.88 - 6.66)	1 vs 3
Ictal headache	0 (0.00)	0 (0.00)	3 (1.50)	0.214	-	-
Post-ictal headache	38 (37.25)	20 (27.03)	16 (8.00)	<0.001^a^;	2.60 (1.85 - 3.64);	1 vs 3;
				<0.001^b^;	2.09 (1.44 - 3.03);	2 vs 3;
				0.127^b^	1.69 (0.86 - 3.31)	1 vs 2

N, number; TTH, tension-type headache; inter-IH, inter-ictal headache; OR, odds ratio; CI, confidence interval; vs, versus; SD, standard deviation; med, median; p25-p75, 25^th^ and 75^th^ percentile; AED, anti-epileptic drug

^a^Multivariable model adjusted for age, sex and anti-migraine therapy

^b^Multivariable model adjusted for age and sex

Figures given as N (%), unless otherwise stated

### Patients with pre-IH vs patients without pre-IH and patients with post-IH vs patients without post-IH

We compared patients with and without pre-IH and patients with and without post-IH (Table 4). Pre-IH was significantly associated with female sex (P = 0.015). Variables significantly associated with post-IH were AEDs polytherapy (P < 0.001), high frequency of seizures (P = 0.002), and tonic-clonic seizures (P = 0.041). Conversely the proportion of patients without post-IH with an AED monotherapy (P = 0.002) and who were seizure-free at the interview (P = 0.012) was significantly higher.

**Table 4:** Comparison of clinical features between patients with and without post-ictal headache and patients with and without pre-ictal headache

	Post-IH	No Post-IH	*p* value	OR (CI 95 %)	Pre-IH	No Pre-IH	*p* value	OR (CI 95 %)
Sample	74 (19.07)	314 (80.93)			26 (6.70)	362 (93.30)		
Males	30 (16.76)	149 (83.24)	0.283	-	6 (3.35)	173 (96.65)	0.015	-
Females	44 (21.05)	165 (78.95)			20 (9.57)	189 (90.43)		
Age *(mean ± SD)*	38.08 ± 13.50	42.00 ± 16.10	0.053	-	38.08 ± 14.54	41.48 ± 15.77	0.286	-
Age at epilepsy onset *(med; p25-p75)*	12; 8-20	15; 8-24	0.135	-	13; 6 - 24	15; 8 - 23	0.601	-
Epilepsy duration *(med; p25-p75)*	21; 12-29	20; 11-32	0.879	-	20.5; 9-32	20.5; 11-32	0.529	-
Epilepsy								
Generalized	18 (24.32)	83 (26.43)	0.710	-	2 (7.69)	99 (27.35)	0.027	-
Focal	55 (74.32)	225 (71.66)	0.645	-	21 (80.77)	259 (71.55)	0.311	-
Unclassified	1 (1.35)	6 (1.91)	0.745	-	3 (11.54)	4 (1.10)	<0.001	-
Frequency of seizures at observation								
Sporadic	23 (31.08)	111 (35.35)	0.487	-	10 (38.46)	124 (34.25)	0.663	-
Monthly/daily	34 (45.95)	85 (27.07)	0.002	-	11 (42.31)	108 (29.83)	0.183	-
Seizure-free	16 (21.62)	116 (36.94)	0.012	-	4 (15.38)	128 (35.36)	0.038	-
Tonic-clonic seizures	53 (71.62)	185 (58.92)	0.041^a^	1.79 (1.02 - 3.12)	16 (61.54)	222 (61.33)	0.983	-
AED therapy								
Monotherapy	23 (31.08)	162 (51.59)	0.002^a^	0.43 (0.25 - 0.74)	13 (50.00)	172 (47.51)	0.806	1.11 (0.50 - 2.45)
Polytherapy	50 (67.57)	138 (43.95)	<0.001^a^	2.64 (1.54 - 4.52)	13 (50.00)	175 (48.34)	0.870	1.07 (0.48 - 2.37)
No therapy	1 (1.35)	14 (4.46)	0.219^a^	0.28 (0.04 - 2.15)	0 (0.00)	15 (4.14)	0.290	-
Photosensitivity	5 (6.76)	18 (5.73)	0.737	-	1 (3.85)	22 (6.08)	0.642	-
Inter-ictal headache	58 (78.38)	130 (41.40)	<0.001	-	23 (88.46)	165 (45.58)	<0.001	-
Migraine	38 (51.35)	64 (20.38)	<0.001^b^	4.06 (2.35 - 7.02)	19 (73.08)	83 (22.93)	<0.001^b^	7.96 (3.20 - 19.80)
Migraine without aura	38 (51.35)	58 (18.47)	<0.001	-	18 (69.23)	78 (21.55)	<0.001	-
Migraine with aura	0 (0.00)	6 (1.91)	0.231	-	1 (3.85)	5 (1.38)	0.325	-
TTH	20 (27.03)	54 (17.20)	0.053	-	3 (11.54)	71 (19.61)	0.311	-

Post-IH, post-ictal headache; Pre-IH, pre-ictal headache; N, number; SD, standard deviation; med, median; p25-p75, 25^th^ and 75^th^ percentile; AED, anti-epileptic drug; TTH, tension-type headache

^a^Multivariable model adjusted for age and sex

^b^Multivariable model adjusted for age, sex and anti-migraine therapy

Figures given as N (%), unless otherwise stated

## Discussion

Headache is a significantly frequent symptom both in patients with epilepsy and in the general population. This is why the prevalence of primary headaches in adult patients with epilepsy remains a matter of debate. Differences in target populations, age range, data collection, diagnostic and classification criteria, methodological study design, and a lower reliability of retrospective studies, may account for such controversial results. Moreover most previous reports focused only on migraine, excluding the other types of primary headache (Table 1) [5–21].

The close collaboration between the Epilepsy and Headache Centers in our study allowed an accurate diagnosis of both epilepsy syndromes and primary headaches.

Among our patients *migraine* was the most frequent type of headache occurring in 26 % of patients, in accordance with previous studies in which the prevalence of migraine ranged from 9 % to 30 %. Although the association between these two diseases has been reported in several epidemiologic studies their relationship has not been clarified yet [5–21]. Previous Ottman and Lipton’s studies on 1948 patients with epilepsy demonstrated a twofold higher risk for migraine in patients with epilepsy compared to their first degree relatives without epilepsy, and they also showed a nearly twofold risk of migraine compared to controls (24 % vs 12 %). However, data from Brodtkorb et al. in a Norwegian population of epilepsy patients failed to confirm a statistically significant association between migraine and epilepsy [6, 26].

Unlike the incidence of migraine we reported *TTH* in only 19 % of our cases, a lower prevalence compared to the general population. To date few studies have addressed TTH and epilepsy and no hypotheses on the relation between the two diseases have been put forward. We could speculate that our result is in line with the type of headache. As a mild condition in spite of seizure disorder, TTH could be underestimated in patients with epilepsy [11, 12, 14, 17, 18, 20, 21].

In any case it is interesting that in our cases both patients with migraine and tension-type *inter-IH* reported seizure-related headache with features similar in quality to their habitual headache attacks.

Analyzing accurately each different types of headaches in relation to seizures (*peri-IH*), we found a slightly lower lifelong prevalence of headaches temporally related to seizures than in the literature (23 % vs 28-50 %) [18]. We actually think it is probably due to the well-controlled epilepsy in most of our patients, with only very rare seizures, or no seizures at all.

*Pre-IH*, usually with migrainous features, was reported in 6 % of our population and only in patients with interictal migraine, in line with previous studies [14, 16, 18, 21, 25]. Only one of our patients with pre-IH had a migraine with aura. In this case the headache attack started about five hours before epileptic seizure onset, and hence did not fall within the diagnostic criteria for *“migralepsy”* that anyway is still a controversial entity [22].

*Ictal headache* occurred only in three patients (0.8 %) without inter-ictal headache. Two of them have temporal lobe epilepsy and the other one a myoclonic epilepsy. All of them reported a tightening quality of headache with a sense of head pressure and throbbing pain during the seizure or a few seconds before. However, as an ictal EEG recording was lacking in these cases, a definition of *“Epileptic Headache”* was not corrected [9, 12, 27]. There is considerable confusion regarding the definition of “Epileptic Headache”, in both headache and epilepsy classifications (ICHD-II and ILAE). The ICHD-II classification (2004) defines “Epileptic Headache” as a headache with migraine features while the patient also has a focal epileptic seizure. These cases are extremely rare, and the term is not used in the current ILAE and ICHD classification [23, 24]. In our study we found that a lower number of adults have ictal headache (recognized as a headache lasting from seconds to days, with evidence of ictal epileptiform EEG discharges) than children. We can speculate that it is strictly due to different symptomatology that children have both in epilepsy and in headache. Children are in fact more likely to have autonomic symptomatology attacks, with long-lasting ictal autonomic manifestations, while adults often have other sensory or motor ictal signs. Thus, it is more probable that many cases are genuine seizures imitating migraine, easily recognizable by an EEG recording [28].

At last in our study *post-IH* was the most frequent type of peri-IH, occurring in 19 % of our patients: 37/74 patients reported a migraine type, 30/74 a TTH, and only in seven cases the quality of headache was unclassified. It is not straightforward to compare our data with previous reports in literature in which post-IH prevalence ranges from 12 % to 52 % [5, 7, 10–14, 16, 18, 20, 21, 29, 30]. Several variables need to be considered. First of all most studies are retrospective [13, 14, 16, 25] and the few prospective studies available were conducted either for a brief period [15] or on pediatric populations [31, 32]. Moreover post-IH prevalence could be influenced by the type and intensity of epilepsy, with a higher prevalence in samples of patients with drug-resistant epilepsy [12, 30]. In our population post-IH was more frequently reported in patients on polytherapy, suggesting a severe epilepsy phenotype, and in patients with a higher seizure frequency (monthly/daily) suggesting that the seizures act as a trigger for headache attacks, as reported in literature [18, 29].

Our study has some limitations. Firstly the retrospective assessment of headaches can lead to a recall bias, especially for peri-IH in patients who had their last seizure years before the interview. For this reason the prevalence of both pre-IH and post-IH may be underestimated in the current study. Secondly we recruited patients from a tertiary care center, thus there is a possible selection bias. However the features of our sample resemble those of population-based epidemiological studies on epilepsy [33]. This is mainly due to the fact that patients with severe encephalopathies were excluded, and the catchment area of our Institute comprised the entire city of Bologna and its hinterland, thus resembling a population. Finally the absence of a control group limits the significance of our results.

Over the last decade, possible pathogenetic mechanisms common to epilepsy and migraine have been investigated in depth. The two disorders seem to be genetically inter-related and are comorbid in several clinical syndromes [34]. An altered membrane channel function and an imbalance between excitatory and inhibitory factors seem to have a central role in the pathogenesis of the two disorders [28, 35–37]. Cortical spreading depression (CSD) is believed to underlie migraine with aura attacks and, according to some evidence, also migraine without aura [28]. Our study disclosed a relatively low occurrence of migraine with aura (1.5 %) that seems not to support this hypothesis. It is likely that both CSD and other mechanisms, such as different environmental or individual factors (genetic or otherwise), are implicated in the link between migraine and epilepsy.

By lowering the trigger threshold in the epileptic focus, CSD could increase the risk of seizures, explaining the onset of pre-IH [2]. Similarly, recurrent seizures may predispose a patient to CSD, inducing post-IH [37]. We suggest that, in analogy with migraine patients, the stressful event represented by an epileptic seizure is a trigger for a headache attack in subjects with migraine or inter-ictal TTH [36, 38, 39]. However, further studies are required to clarify the mechanisms underlying the two conditions.

## Conclusion

Half of our patients with epilepsy presented either inter-ictal or peri-ictal headaches or both, confirming the bidirectional relationship between these two pathologies. Migraine is the most prevalent type of headache. Patients presenting migraine or tension-type inter-IH seem to be more readily predisposed to develop a seizure-related headache with features similar to their habitual headache attacks. However, only patients with inter-ictal migraine appear to be very prone to present a migraine headache before seizures. Further population studies are required to establish whether comorbidity exists between epilepsy and migraine, or whether it is a chance association between two relatively common neurological disorders. In addition, prospective studies including the compilation of a headache and seizure diary may serve to establish if one disease represents a risk factor for the other. It is crucial to explore this association and identify clinical subgroups in both epilepsy and headache patients sharing common pathogenic pathways and possibly common therapeutic targets.

## Abbreviations

Inter-IH: Inter-ictal Headache
Peri-IH: Peri-ictal Headache
ICHD-2: International Classification of Headache Disorders
TTH: Tension-type Headache
Pre-IH: Pre-ictal Headache
Post-IH: Post-ictal Headache
ILAE: International League Against Epilepsy
AED: Anti-epileptic Drug
CSD: Cortical Spreading Depression
OR: Odd Ratio
